# Tubular epithelial C1orf54 mediates protection and recovery from acute kidney injury

**DOI:** 10.1111/jcmm.13765

**Published:** 2018-07-12

**Authors:** Hongyang Xie, Yaqiong Wang, Hang Zhang, Qin Fan, Daopeng Dai, Lingfang Zhuang, Rong Tao, Qiujing Chen, Weifeng Shen, Lin Lu, Xiaoqiang Ding, Ruiyan Zhang, Xiaoxiang Yan

**Affiliations:** ^1^ Department of Cardiology Rui Jin Hospital Shanghai Jiaotong University School of Medicine Shanghai China; ^2^ Institute of Cardiovascular Diseases Shanghai Jiaotong University School of Medicine Shanghai China; ^3^ Department of Nephrology Zhongshan Hospital Fudan University Shanghai China

**Keywords:** C1orf54, cell proliferation, ischaemia‐reperfusion injury, PI3K/AKT

## Abstract

Acute kidney injury (AKI) incidence among hospitalized patients is increasing steadily. Despite progress in prevention strategies and support measures, AKI remains correlated with high mortality, particularly among ICU patients, and no effective AKI therapy exists. Here, we investigated the function in kidney ischaemia‐reperfusion injury (IRI) of C1orf54, a newly identified protein encoded by an open reading frame on chromosome 1. C1orf54 expression was high in kidney and low in heart, liver, spleen, lung and skeletal muscle in healthy mice, and in the kidney, C1orf54 was expressed in tubular epithelial cells (TECs), but not in glomeruli. C1orf54 expression was markedly decreased on Day 1 after kidney IRI and then gradually recovered to baseline levels by Day 7. Notably, relative to wild‐type mice, C1orf54‐knockout mice exhibited impaired TEC proliferation and delayed recovery after kidney IRI, which led to deteriorated renal function and increased mortality. Conversely, adenovirus‐mediated C1orf54 overexpression promoted TEC proliferation and ameliorated kidney pathology, which resulted in accelerated renal repair and improved renal function. Mechanistically, C1orf54 was found to promote TEC proliferation through PI3K/AKT signalling. Thus, C1orf54 holds considerable potential as a therapeutic target in kidney IRI.

## 1 INTRODUCTION

Acute kidney injury (AKI) has been described as “[A]n abrupt (within 48 hours) reduction in kidney function” as measured by serum creatinine increases.^1^ AKI incidence has risen steadily in several demographic groups, particularly in the context of multiorgan disease and sepsis. Despite advances in preventive strategies and support measures, AKI remains associated with high morbidity and mortality, generally reported to be in the 30%‐70% range. Moreover, even if AKI patients survive the acute illness, they face chronic consequences, including high risk of developing or exacerbating chronic kidney disease and accelerated development of end‐stage renal disease.^2^, ^3^

Renal ischaemia‐reperfusion injury (IRI), a common AKI cause, results from a generalized or localized impairment of oxygen and nutrient delivery to, and waste product removal from, kidney cells.^4^ Local tissue oxygen supply and demand and accumulation of metabolism waste products are mismatched, and this results in tubular epithelial cell (TEC) injury, which, if severe, causes cell death by apoptosis and necrosis (acute tubular necrosis), coupled with organ‐level functional impairment of water/electrolyte homeostasis and reduced excretion of metabolism waste products.^4^, ^5^ However, because the mechanism underlying kidney IRI is largely unknown, a treatment strategy is lacking.

The kidney can repair itself,^5^ and a crucial pathologic feature of post‐AKI repair is renal TEC proliferation and regeneration.^6^ Cell proliferation repairs the damaged kidney by replacing TECs lost due to cell death.^4^ Accumulating evidence indicates that paracrine signalling from endogenous surviving epithelial cells (eg distal TECs) could underlie anti‐IRI effects, and multiple factors secreted from distal nephrons could produce paracrine effects to promote the proliferation and repair of surviving tubular cells through cell‐to‐cell crosstalk.^4^, ^6^

Chromosome 1 is the largest human chromosome and contains ~8% of all human genetic information and thus might be more representative of the human genome than other chromosomes.^7^ Chromosome 1 harbours *C1orf54*, which encodes a predicted secreted protein of mostly unknown function. As a secreted protein, we proposed that C1orf54 may have some function in the physiological or diseased states. Thus, we generated C1orf54 deficiency mice to examine its role in diseased state. In this study, we demonstrated that C1orf54 was expressed exclusively in renal TECs, and by gain and loss of function studies, we revealed that C1orf54 promoted renal repair and TEC proliferation through PI3K/AKT signalling, which alleviated kidney damage after IRI.

## 2 MATERIALS AND METHODS

### 2.1 Mice

Male 8‐ to 10‐week‐old C1orf54^−/−^ (C1 KO) mice had been backcrossed to C57BL/6 at least 10 generations before use. Mice were housed in individual microisolator cages with free access to sterile acidified water and irradiated food in a specific‐pathogen‐free facility. This study and all animal procedures conformed to the Guide for the Care and Use of Laboratory Animals, published by the US National Institutes of Health (NIH publication no. 85‐23, revised 1996), and were approved by the Animal Care Committee of Shanghai Jiaotong University School of Medicine.

### 2.2 Generation of *C1orf54*‐knockout mice

C1 KO mice were developed by Shanghai Model Organisms Center, Inc. (Shanghai, China). To generate C1orf54‐floxed mice, a *C1orf54*‐targeting vector was constructed with ET cloning techniques in EL250 bacterial cells; the construct was designed to flank exon 3 with loxp sites and a pGK‐neomycin‐polyA cassette. The vector was electroporated into B6/129 embryonic stem (ES) cells, which were then selected with 2 drugs, G418 and ganciclovir, to screen for homologous recombination clones. Long PCR and sequencing were used to identify and confirm the ES clones exhibiting correct homologous recombination, which was genotyped with these primers: 5′‐ACCCTTGGTGTCTATGCTGGTC‐3′ and 5′‐CTGGAAGATGTCCGTGGTGTTA‐3′, for correct 5′‐homology‐arm recombination; and 5′‐CAAAGAGGGTGAGAAGGTAAGC‐3′ and 5′‐CAGACATCAATAGGAGCAGGAAT‐3′, for correct 3′‐homology‐arm recombination. Positive ES cell clones were expanded and microinjected into C57BL/6J blastocysts to generate chimeric mice, which were crossed with C57BL/6J mice to obtain *C1orf54*‐floxed heterozygous (*C1orf54* ^*flox/+*^) mice. The *C1orf54* ^*flox/+*^ mice were mated with EIIA‐Cre mice (*Tg* (*EII*a‐Cre); The Jackson Laboratory) expressing Cre recombinase in the early embryo to obtain heterozygous *C1orf54*‐knockout (*C1orf54* ^*+/−*^) mice, which were intercrossed to generate the homozygous *C1orf54*‐knockout (*C1orf54* ^*−/−*^) mice used here.

### 2.3 AKI induction in mice

Kidney IRI was induced as described.^8^ Mice were anesthetized by intraperitoneally injecting sodium pentobarbital (50 mg/kg bodyweight; Sigma‐Aldrich, St. Louis, USA) before surgery, during which both kidneys were exposed through a flank incision and the kidney pedicles were clamped for 30 minutes at 37°C by a heat device. Sham‐operated animals underwent the same procedure except for the renal pedicle clamping. For the histological analysis of kidney from sham‐operated mice, we killed the mice on Day 7 after operation.

### 2.4 Histology and Immunohistochemistry

Kidneys were fixed in 4% paraformaldehyde (24 hours) and embedded in paraffin, and then, 5‐μm sections were subject to PAS and Masson's trichrome staining as per standard protocols. At least 10 random fields from each sample were analysed. Immunohistochemical staining was performed with anti‐C1orf54 (Sigma‐Aldrich, St. Louis, USA) as described,^9^ and C1orf54‐positive cells were counted under a microscope (Olympus, 400× magnification).

For immunofluorescence analysis, tissue sections were boiled in citrate buffer solution (10 minutes), treated with 0.2% Triton X‐100 (30 minutes, room temperature) and incubated overnight with rabbit anti‐C1orf54 (H00079630‐B04P, Sigma‐Aldrich) and rabbit monoclonal anti‐Ki‐67 (9129s, Cell Signaling Technology) to detect C1orf54 and proliferation, respectively. Villin antibody (Santa Cruz) was used to colocalize the C1orf54 and renal tubular. All sections were incubated (1 hour) with donkey anti‐rabbit Alexa Fluor^®^ 488‐conjugated secondary antibody (A‐21206, Invitrogen, Carlsbad, CA, USA) and donkey antimouse Alexa Fluor^®^‐555 conjugated secondary antibody (A‐31570, Invitrogen), costained with DAPI and imaged under an Olympus microscope.

### 2.5 Renal‐damage evaluation

The areas of PAS‐stained debris (at corticomedullary junction) and brush borders (at corticomedullary junction and cortex region) in kidney specimens were quantified with NIH ImageJ software, and the percentages of these areas relative to the entire section in a slide are presented; 5 kidney sections from at least 3 mice from each group were examined. Specimens were evaluated in a manner blinded to the mouse strain.

### 2.6 Serum BUN and creatinine measurement

At the end of experiments, mice were anesthetized, and blood was collected from the retro‐orbital plexus. Serum creatinine (Cre) and BUN concentrations were measured with creatinine serum kit (2 whole plate kit, Arbor Assays, KB02‐H1) and urea nitrogen (BUN) detection kit (2 plates, Arbor Assays, K024‐H1).

### 2.7 TUNEL assay

To examine apoptosis in damaged kidneys from WT and C1 KO mice post‐IRI, we used an in situ cell death detection kit (Roche, 11684795910) for TUNEL (terminal deoxynucleotidyl transferase dUTP nick‐end labelling) staining of apoptotic cells in renal sections. TUNEL^+^ cells were counted by researchers blinded to the mouse groups.

### 2.8 BrdU incorporation

A BrdU labelling reagent (ThermoFisher, 000103; 1 mL/100 g) was intraperitoneally injected into C1 KO and WT mice, and 2 hours later, the mice were killed and kidneys were harvested. Subsequently, kidney paraffin sections were costained with a BrdU monoclonal antibody (Thermo Fisher, MA5‐11285) and DAPI.

### 2.9 Cell culture

HK‐2 cells were cultured in DMEM/F12 supplemented with 10% FBS, penicillin and streptomycin. To mimic IRI in vitro, cells in FBS‐free medium were exposed to hypoxia (1% O_2_, 94% N_2_ and 5% CO_2_) for 6 hours at 37°C and then cultured under normoxia (5% CO_2_, 95% air) for reoxygenation (for 0, 6, 12 or 24 hours) and harvested for analyses. HK‐2 cells were transiently transfected with negative control or C1orf54‐specific siRNA (100 nmol/L per 10^5^ cells) by Lipofectamine (Invitrogen, Carlsbad, CA, USA).

### 2.10 Adenovirus, gene transfer

Recombinant adenoviruses were generated by subcloning the C1orf54 cDNA into pShuttle‐CMV vector and then produced by following the AdEAsy XL adenoviral vector system protocol (Agilent Technologies, Santa Clara, CA, USA). Cultured cells were infected by adding viruses (5 MOI) in serum‐containing culture medium; after 2 hours, cultures were washed with PBS and incubated with fresh medium. For adenovirus‐mediated gene transfer in vivo, hydrodynamic injection was used; adenoviruses in 1 mL of PBS were injected within 10 seconds into the mouse tail vein 24 hours before treatment. To block PI3K/AKT pathway, wortmannin (PI3K inhibitor, 1.0 mg/kg) was intraperitoneally injected into the C1orf54 overexpressing mice once every other day.

### 2.11 Western blotting

Total protein extracts were prepared from renal tissues or cells, and after measuring protein concentrations (BCA method), samples were electrophoresed and transferred to PVDF membranes, which were blocked (5% milk/TBST, room temperature) and then incubated (overnight, 4°C) with these primary antibodies (in 3% BSA solution): goat anti‐C1orf54 (sc‐240106, Santa Cruz); mouse monoclonal anti‐C1orf54 (H00079630‐B04P, Abnova Biotechnology); caspase‐3 and cleaved caspase‐3 antibodies (9662 and ASP175, respectively); mouse monoclonal anti‐PCNA (PC10, 2586s); AKT and phospho‐AKT antibodies (9272s, 9271s); p38‐MAPK and phospho‐p38‐MAPK antibody (8690s, 9211s); β‐catenin antibody (8480s); SAPK/JNK and phospho‐SAPK/JNK antibody (9252s, 9251s); STAT3 and phospho‐STAT3 antibody (9139s, 9145s), Cell Signaling Technology; after secondary antibody staining (room temperature, 1 hour), bands were quantified with ImageJ densitometry software.

### 2.12 Statistical analyses

Power was not calculated to predetermine sample sizes, and randomization was not used to determine samples or mice to be allocated to experiments. Areas were calculated in a blinded manner. In vitro experiments were repeated at least thrice. Data were analysed with GraphPad Prism (GraphPad Software Inc., San Diego, CA) and are presented as means ± SD unless specified otherwise. Paired results were assessed with parametric tests (eg Student's *t* test). Multiple groups were compared with 1‐way or 2‐way ANOVA followed by Bonferroni's *post hoc* test. For Kaplan‐Meier curves, *P* values were determined with the log‐rank test.

## 3 RESULTS

### 3.1 C1orf54 was highly expressed in renal TECs and down‐regulated after kidney IRI

**Figure 1:**
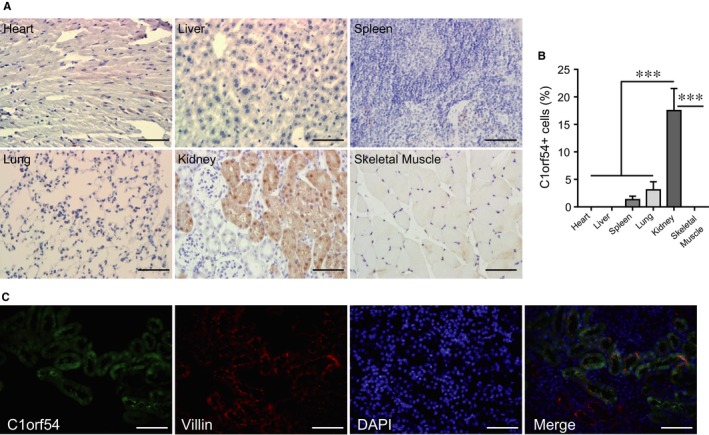
C1orf54 was highly expressed in renal tissue. A, Immunohistochemical staining of C1orf54 in sections of heart, liver, spleen, lung, kidney and skeletal muscle. Scale bar, 100 μm. B, Areas of C1orf54‐positive cells, as shown in (A), were quantified (n = 6). C, Double staining of C1orf54 and villin in the kidney. Scale bar, 100 μm. Data are expressed as means ± SD for each group. ***P <* *.001, vs heart, liver, spleen, lung and skeletal muscle

No C1orf54 function in the body has been reported previously. Here, we began by examining C1orf54 expression in different organs and tissues in healthy mice: Immunohistochemical staining (Figure 1A,B) revealed for the first time that C1orf54 expression was high in the kidney but low in heart, liver, spleen, lung and skeletal muscle and that renal C1orf54 was expressed in TECs but not glomeruli (Figure 1A). Unexpectedly, C1orf54 was shown to be highly expressed in small intestine (Figure S1A). Furthermore, double stainings with C1orf54 and villin confirmed that C1orf54 was exclusively in TECs, and specifically, it localized in both nucleus and cytoplasm (Figure 1C).

**Figure 2:**
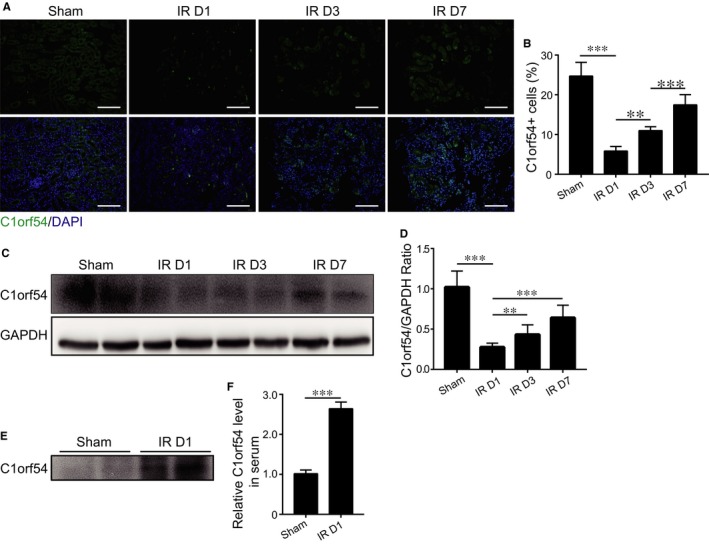
C1orf54 expression was transiently decreased after kidney ischaemia‐reperfusion injury (IRI). A, Immunofluorescent staining of C1orf54 at different time‐points after kidney IRI. Scale bar, 100 μm. B, Quantification of C1orf54‐positive cells (n = 6). C, Western blotting analysis of C1orf54 expression in renal tissues post‐IRI and D, quantification of C1orf54 expression relative to GAPDH (n = 6). E, Western blotting examination of C1orf54 levels in the serum of WT mice on Day 1 after IR surgery and F, quantification of band densities (n = 4). Data are expressed as means ± SD. ***P <* *.001, **P <* *.01

TECs respond to ischaemic insults: In AKI, TECs undergo cell death and proliferation for repair.^4^, ^6^ Thus, we used the kidney IRI model to investigate C1orf54 function in the mouse kidney. Immunofluorescence staining of kidney sections revealed a marked decrease in C1orf54 expression on Day 1 post‐IRI, followed by gradual recovery to baseline levels by Day 7 (Figure 2A,B). Western blotting results confirmed the temporal dynamics of renal C1orf54 expression (Figure 2C,D). Moreover, C1orf54 released from TECs and localized around tubules after kidney IRI (Figure 2A). Thus, serum C1orf54 level was ~3‐fold higher after kidney IRI than after sham operation (Figure 2E,F).

### 3.2 C1orf54 deficiency exacerbated renal dysfunction after kidney IRI

**Figure 3:**
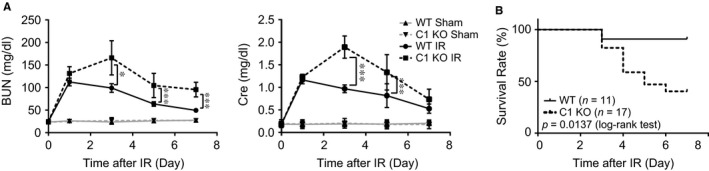
C1orf54 deficiency aggravated renal dysfunction after kidney IRI. A, Serum levels of blood urea nitrogen (BUN) and creatinine (Cre) were determined at different time‐points after kidney IRI (n = 6). Data are expressed as means ± SD. ***P <* *.001, *P <* *.05. B, Survival of WT and C1orf54‐knockout (C1 KO) mice post‐IRI

To assess C1orf54's pathophysiological role in kidney IRI, we generated C1orf54‐knockout (C1 KO) mice (Figure S1B‐D). C1 KO mice developed similarly as wild‐type (WT) mice and showed no difference at baseline in 2 renal dysfunction markers: serum blood urea nitrogen (BUN) and creatinine (Figure 3A). In WT mice, the 2 markers peaked on Day 1 post‐IRI and then gradually recovered, whereas in C1 KO mice, the markers peaked on Day 3, were substantially higher than those in WT mice and did not reach baseline even on Day 7 post‐IRI (Figure 3A). Accordingly, the survival rate of C1 KO mice was significantly lower than that of WT mice (60% vs 90%, log‐rank test, *P *= .0137) (Figure 3B). These results indicated that C1orf54 deficiency impaired and delayed the post‐IRI renal recovery process.

**Figure 4:**
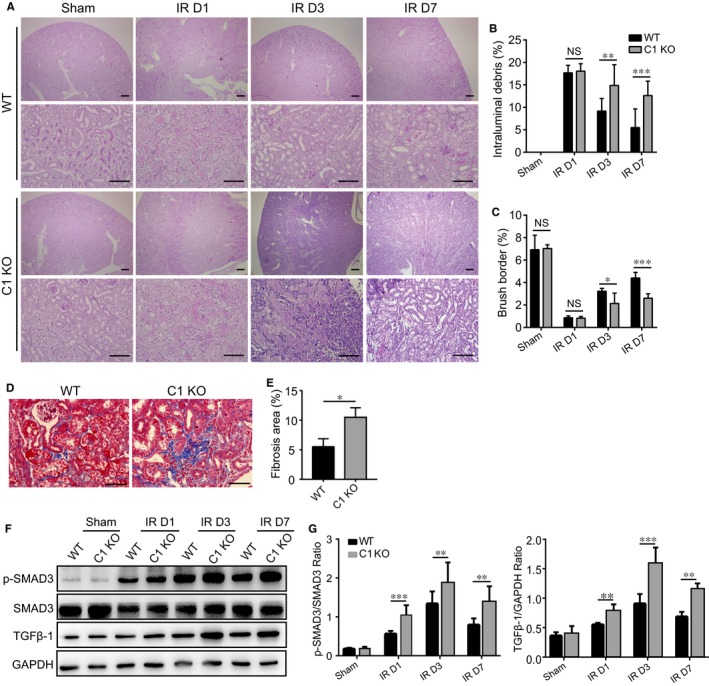
Histological analysis of the kidney after IRI in WT and C1 KO mice. A, Histological PAS staining of the kidney from WT and C1 KO mice at different time‐points after kidney IRI. Scale bars, 400 and 200 μm, upper and lower images. B, C, Quantification of areas of intraluminal debris and brush borders at the corticomedullary junction (n = 4). D, Masson's trichrome staining of the kidney on Day 14 after IRI. Scale bars, 200 μm. E, Fibrosis area in WT and C1 KO mice. F, G, Western blotting analysis of p‐SMAD3/SMAD3 and TGFβ‐1 levels in the kidney of WT and C1 KO mice at different time‐points after IRI (n = 4). Data are expressed as means ± SD. NS, not significant; ***P <* *.001, **P <* *.01, *P <* *.05

Next, we stained renal sections with periodic acid‐Schiff (PAS) to examine how WT and C1 KO mice differed in 2 renal pathologic indicators: intraluminal debris and brush borders (Figure 4A‐C). On Day 1 post‐IRI, the kidney TEC structure was impaired in both WT and C1 KO mice; dilation of numerous tubules at the corticomedullary junction, congestion with necrotic cells and loss of nuclei and brush borders were observed. However, starting on Day 3, PAS^+^ intraluminal debris levels were significantly lower in WT mice than in C1 KO mice, whereas the brush‐border extent was higher in TECs of WT mice than that of C1 KO mice. Moreover, even on Day 7, C1 KO mice exhibited no clearance of PAS^+^ intraluminal debris or brush‐border recovery at the corticomedullary junction. Accordingly, Masson's trichrome staining revealed greater renal fibrosis in C1 KO mice than in WT mice on Day 14 post‐IRI (Figure 4D,E). In line with these findings, phosphorylation of SMAD3 and expression of TGF‐β1 were greatly enhanced in C1 KO mice after kidney IRI (Figure 4F,G), indicating that TGF‐β/Smad3 signalling is a key pathway in renal fibrosis associated with C1orf54 deficiency in kidney.

### 3.3 C1orf54 promoted renal TEC proliferation

**Figure 5:**
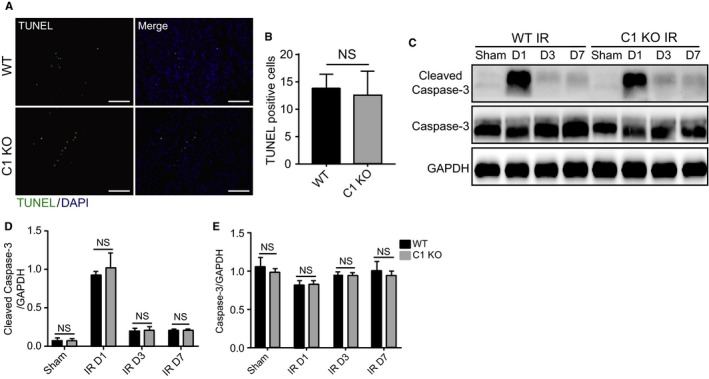
C1orf54 loss did not affect apoptosis after kidney IRI. A, TUNEL staining of renal sections from WT and C1 KO mice on Day 1 after IRI. Scale bars, 100 μm. B, Quantification of TUNEL^+^ cells (n = 6). C‐E, Western blotting analysis of cleaved caspase‐3 and caspase‐3 levels in damaged kidneys at different time‐points after IRI (n = 6). Data are expressed as means ± SD. NS, not significant

To precisely examine C1orf54's involvement in renal recovery, we first tested whether C1orf54 affected cell death. As expected, apoptosis or necrosis occurred immediately after hypoxia injury,^10^ but unexpectedly, we detected no difference in the number of TUNEL^+^ cells and the levels of cleaved caspase‐3 and caspase‐3 between WT and C1 KO mice on Day 1 post‐IRI (Figure 5). These results indicated that C1orf54 did not affect TEC apoptosis.

**Figure 6:**
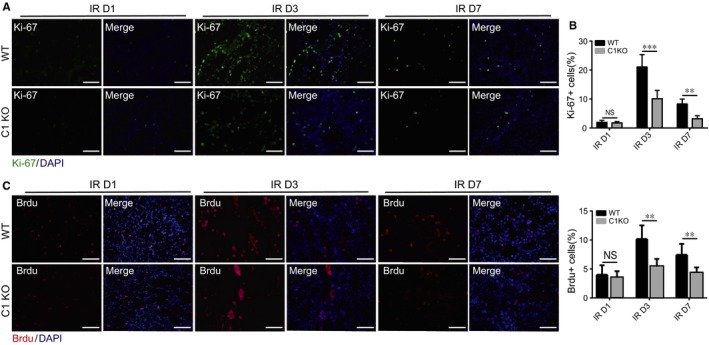
Absence of C1orf54 impaired tubular epithelial cell (TEC) proliferation. A, Ki‐67 immunofluorescent staining at the corticomedullary junction in renal sections from WT and C1 KO mice at indicated time‐points after kidney IRI. Scale bars, 100 μm. B, Quantification of Ki‐67^+^ cells (n = 6). C, At different time‐points after IRI, BrdU was intraperitoneally injected into WT and C1 KO mice, and 2 h after injection, kidney tissues were harvested for BrdU immunofluorescent staining. Scale bars, 100 μm. D, Quantification of BrdU^+^ cells (n = 6). Data are expressed as means ± SD. NS, not significant; ***P <* *.001, **P <* *.01

Because post‐injury TEC proliferation is critical for recovery from AKI,^11^ we tested whether C1orf54 deficiency impaired this proliferative response. Proliferation is an immediate early response that peaks at 24‐48 hours post‐IRI and declines thereafter, concurrently with morphological recovery of tubular structures.^11^, ^12^ We stained C1 KO and WT kidney sections at various times post‐IRI for Ki‐67, a cell proliferation marker (Figure 6A,B): Ki‐67^+^ cells were mainly located at the corticomedullary junction in WT and C1 KO mice, and their numbers did not differ significantly on Day 1 post‐IRI; however, from Day 3 onwards, markedly fewer Ki‐67^+^ cells were detected in C1 KO mice than in WT mice. Moreover, analysis of another proliferation indicator, bromodeoxyuridine (BrdU) incorporation, confirmed diminished TEC proliferation at the corticomedullary junction in C1 KO mice relative to that in WT mice on Days 3 and 7 post‐IRI (Figure 6C,D).

### 3.4 C1orf54 was required for hypoxia/re‐oxygenation‐induced TEC proliferation

**Figure 7:**
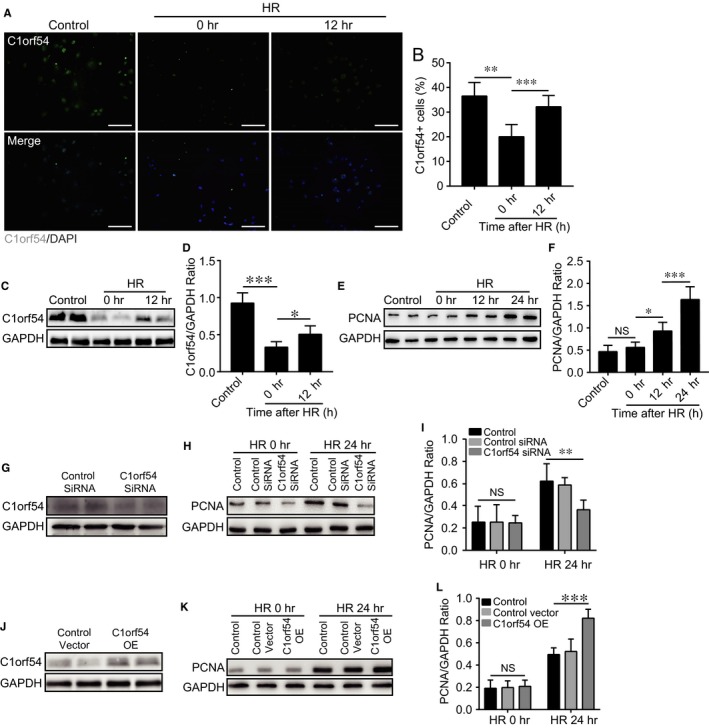
C1orf54 was required for hypoxia‐induced TEC proliferation. HK‐2 cells were subject to hypoxia treatment for 6 h followed by reoxygenation (HR) for different periods. A, Immunofluorescent staining of C1orf54 in HK‐2 cells and B, quantification of C1orf54‐positive cells (n = 6) after 6‐h hypoxia treatment and reoxygenation for different times. Scale bars, 100 μm. C‐F, Western blotting analysis of C1orf54 and PCNA levels in HK‐2 cells harvested at indicated time‐points after reoxygenation (n = 6). G, Western blotting results showing efficient knockdown of C1orf54 in HK‐2 cells transfected with C1orf54‐targeting siRNA but not control siRNA (n = 4). H, I, Reduction of PCNA expression after HR stress in HK‐2 cells transfected with C1orf54 siRNA (n = 6). J, Western blotting results showing C1orf54 overexpression in HK‐2 cells infected with C1orf54‐overexpressing adenovirus (C1orf54 OE) but not control adenovirus. K, L, PCNA up‐regulation in C1orf54‐overexpressing HK‐2 cells at 24 h after HR (n = 6). Data are expressed as means ± SD. NS, not significant; ***P <* *.001, **P <* *.01, *P <* *.05

We examined the mechanism underlying C1orf54‐mediated TEC proliferation by performing in vitro studies on a tubule epithelial cell line: We assessed HK‐2 cell proliferation after 6‐hour hypoxia treatment followed by reoxygenation for different periods. Immunofluorescence staining (Figure 7A,B) and Western blotting (Figure 7C,D) results confirmed that C1orf54 levels decreased following hypoxia and then recovered after 12‐hour reoxygenation; moreover, in line with previous findings,^13^ hypoxia/reoxygenation up‐regulated the cell proliferation marker PCNA (Figure 7E,F).

Next, through siRNA‐mediated knockdown, C1orf54 was down‐regulated in HK‐2 cells (Figure 7G), and in these cells, hypoxia/reoxygenation‐induced PCNA expression was also significantly decreased (Figure 7H,I). Conversely, adenovirus‐mediated C1orf54 overexpression markedly enhanced PCNA expression in HK‐2 cells (Figure 7J‐L). Thus, C1orf54 was essential for hypoxia/reoxygenation‐induced TEC proliferation.

### 3.5 C1orf54 promoted TEC proliferation through PI3K/AKT signalling

**Figure 8:**
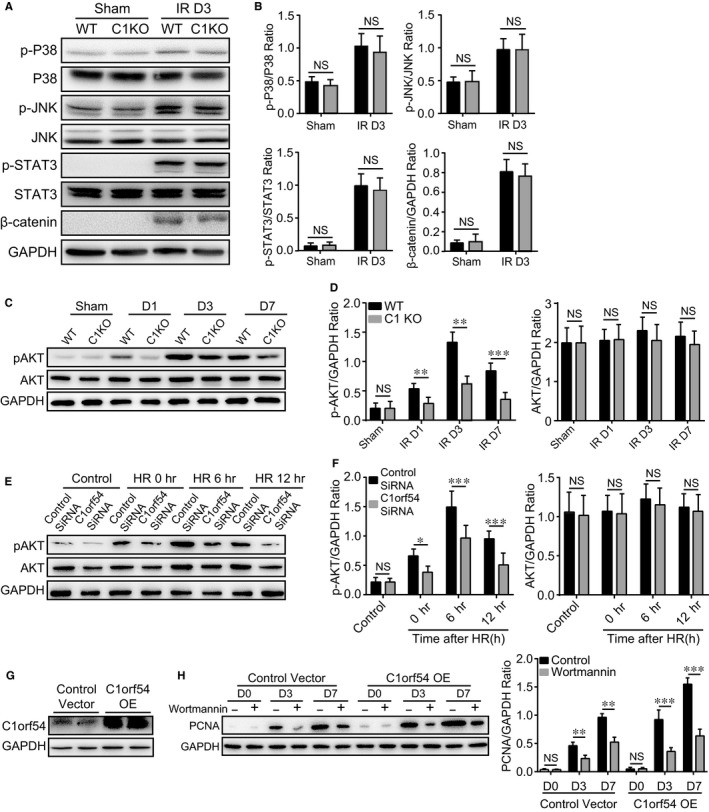
C1orf54 promoted TEC proliferation through PI3K/AKT signalling. A, B, Western blotting analysis of p‐P38/P38, p‐JNK/JNK, p‐STAT3/STAT3 and β‐catenin levels in the kidney of WT and C1 KO mice at Day 3 after IRI (n = 4). C, D, Western blotting analysis of p‐AKT/AKT levels in the kidney of WT and C1 KO mice at different time‐points after IRI (n = 6). E, F, HK‐2 cells were transfected with control siRNA or C1orf54 siRNA and then subject to 6‐h hypoxia treatment and reoxygenation (HR) for different periods. Western blotting was performed to examine phospho‐AKT (p‐AKT) and AKT levels (n = 6). G, H, Control and C1orf54 adenoviruses were injected through the tail vein 24 h before kidney IRI, and the PI3K/AKT‐signalling inhibitor wortmannin was injected daily through the peritoneum. C1orf54 adenoviruses injection greatly increased its protein expression in the kidney in vivo (G), PCNA expression (H) was examined at different time‐points after IRI (n = 6). Data are expressed as means ± SD. NS, not significant; ***P <* *.001, **P <* *.01, *P <* *.05

P38 MAPK, JNK, STAT3, Wnt/β‐catenin and PI3K/AKT signalling pathways were reported to be involved in TEC proliferation^11^, ^12^; thus, we examined whether these signalling pathways participate in C1orf54‐mediated TEC proliferation. We found that kidney IRI led to P38, JNK, STAT3 and β‐catenin activation, while deficiency of C1orf54 did not alter phosphorylation of P38, JNK and STAT3, and expression level of β‐catenin (Figure 8A,B), indicating that these signalling pathways were not essential for TEC proliferation. Next, we revealed that IRI‐induced AKT phosphorylation was considerably lower in C1 KO mice than in WT mice (Figure 8C,D); accordingly, C1orf54 knockdown significantly decreased hypoxia/reoxygenation‐induced AKT phosphorylation in vitro (Figure 8E,F). To examine whether PI3K/AKT signalling is required for C1orf54‐induced TEC proliferation, we used the PI3K inhibitor wortmannin in vivo to block PI3K/AKT signalling. We first confirmed that adenovirus‐mediated C1orf54 overexpression in vivo greatly increased C1orf54 expression in the kidney (Figure 8G). C1orf54 overexpression markedly increased PCNA expression, and this was potently suppressed by wortmannin (Figure 8H). These results indicated that C1orf54 induced TEC proliferation through PI3K/AKT signalling.

### 3.6 C1orf54 overexpression alleviated post‐IRI renal dysfunction

**Figure 9:**
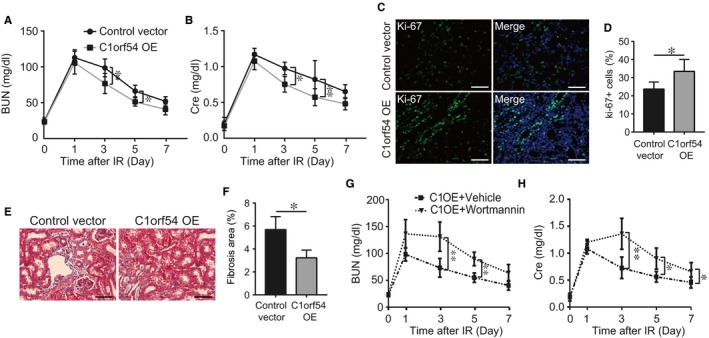
C1orf54 overexpression promoted renal recovery after kidney IRI. WT mice were transfected with C1orf54‐overexpressing adenovirus or control vector and then subject to IRI. A, B, BUN and Cre levels after kidney IRI (n = 6). C, D, Ki‐67 immunofluorescent staining in renal sections from control and C1orf54‐overexpressing mice on Day 3 post‐IRI. Ki‐67^+^ cells were quantified (n = 6). Scale bars, 100 μm. E, F, Histological analysis of renal sections from control and C1orf54‐overexpressing mice on Day 14 after IRI. Fibrotic areas were quantified (n = 6). G, H, WT mice were transfected with C1orf54‐overexpressing adenovirus, and wortmannin (PI3K inhibitor) was intraperitoneally injected into the mice once every other day, and serum BUN and Cre levels were examined at different time‐points after kidney IRI (n = 6). Data are expressed as means ± SD. **P <* *.01, *P < .05

Lastly, we examined whether C1orf54 helps mitigate IRI‐induced renal dysfunction. C1orf54 overexpression significantly decreased serum BUN and creatinine starting from Day 3 post‐IRI (Figure 9A,B) and increased Ki‐67^+^ cell numbers (Figure 9C,D) and alleviated renal fibrosis (Figure 9E,F) post‐IRI. However, blockade of PI3K/AKT pathway with wortmannin prevented the protective effect of C1orf54 on renal function after kidney IRI (Figure 9G,H). These results suggested that C1orf54 overexpression enhanced TEC proliferation and improved renal function via PI3K/AKT signalling after kidney IRI.

## 4 DISCUSSION

This study demonstrated that C1orf54 was critically involved in post‐IRI renal repair: Whereas C1orf54 deficiency led to suppressed TEC proliferation, delayed repair and aggravated kidney dysfunction, C1orf54 overexpression increased TEC proliferation, accelerated repair and improved kidney function, mainly by activating PI3K/AKT signalling.

After AKI, renal TECs undergo complex pathological repair processes, including migration, proliferation and redifferentiation.^14^ For kidney repair, “cell‐to‐cell crosstalk” and growth factors are reported to be essential, and secreted proteins could function as growth factors that facilitate tissue repair and regeneration.^15^, ^16^, ^17^ Approximately 2000 proteins in the proteome have been reported to possess the structural features necessary for secretion into the extracellular milieu, but the biological functions of these proteins are largely unknown. Recently, the bone marrow cell secretome was screened, and after bioinformatic analysis was used to eliminate genes characterized or predicted to encode potential non‐secreted proteins, 150 genes were analysed^18^; 2 proteins, C19orf10 (“myeloid‐derived growth factor”) and C1orf54, were found to possess potential secreted protein functions, and further investigation revealed that C19orf10 promoted angiogenesis and cardiomyocyte survival after myocardial infarction. However, C1orf54 function remains relatively unknown, and few studies have reported its role in any disease. We demonstrated that C1orf54 was highly expressed in renal TECs and involved in TEC proliferation, accelerated repair and improved kidney function after renal IRI. Further studies are needed to examine whether C1orf54 could act as a biomarker for acute tubular injury and excrete in the urine.

The kidney IRI model is commonly used to study the AKI mechanism. By this model together with C1orf54 deletion and adenovirus‐mediated overexpression, we demonstrated that C1orf54 was essential for TEC proliferation and kidney repair, but did not affect TEC apoptosis. C1orf54 was reported to potentially function in the pathophysiology of diffuse congenital hyperinsulinism through the IGF‐1/mTOR/AKT pathway,^19^ and the PI3K/AKT pathway is known to play a critical role in cell proliferation.^20^, ^21^ Here, we demonstrated that AKT phosphorylation was inhibited in C1orf54‐deficient mice after kidney IRI and that this could be reproduced in HK‐2 cells subject to hypoxia/reoxygenation.

In the present study, on Day 5 post‐IRI, about 50% of the C1orf54 KO mice were dead. However, kidney function as indicated by BUN and creatinine already started to drop on Day 3, which points to an additional risk factor beside AKI in the KO mice. This paradox may be due to the following: First, despite the gradual recovery of renal function, but it has not returned to normal, there is still a serious renal insufficiency; Second, acute renal insufficiency is often complicated with other organ injuries, namely multiple organ dysfunction, which may result in its death later after kidney ischaemia injury.

We suspect that C1orf54 acts as a protein that is secreted from TECs damaged after kidney IRI. Immunofluorescence imaging revealed certain C1orf54‐positive particles around surviving and necrotic tubules, and Western blotting results showed that serum C1orf54 levels were low in sham‐operated mice and markedly increased in mice subject to kidney IRI. However, further investigation is required to ascertain how C1orf54 is released from TECs and how C1orf54 regulates PI3K/AKT signalling.

In conclusion, this study demonstrated for the first time that C1orf54 was expressed exclusively in renal TECs and promoted TEC proliferation and kidney repair post‐IRI by acting through the PI3K/AKT signalling pathway. Therefore, our findings identify a potential target for AKI treatment.

## CONFLICT OF INTEREST

The authors confirm that there are no conflict of interests.
