# Supplementary figures and images for: Tubular epithelial C1orf54 mediates protection and recovery from acute kidney injury
Source: J Cell Mol Med. 2018 Jul 12;22(10):4985–96. doi: 10.1111/jcmm.13765 (PMC6156286; doi:10.1111/jcmm.13765)

# Supplementary Figure 1

A

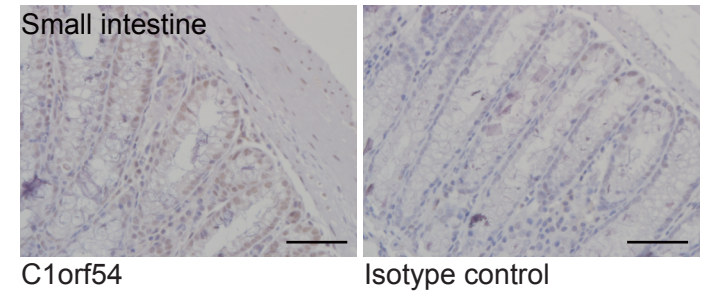

B

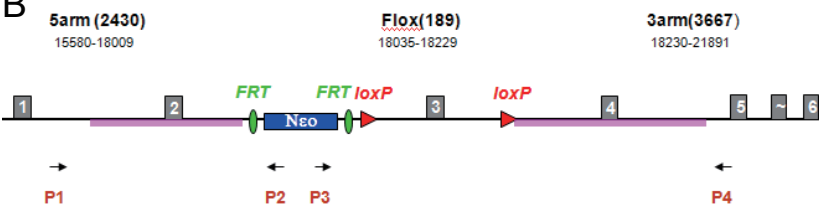

C

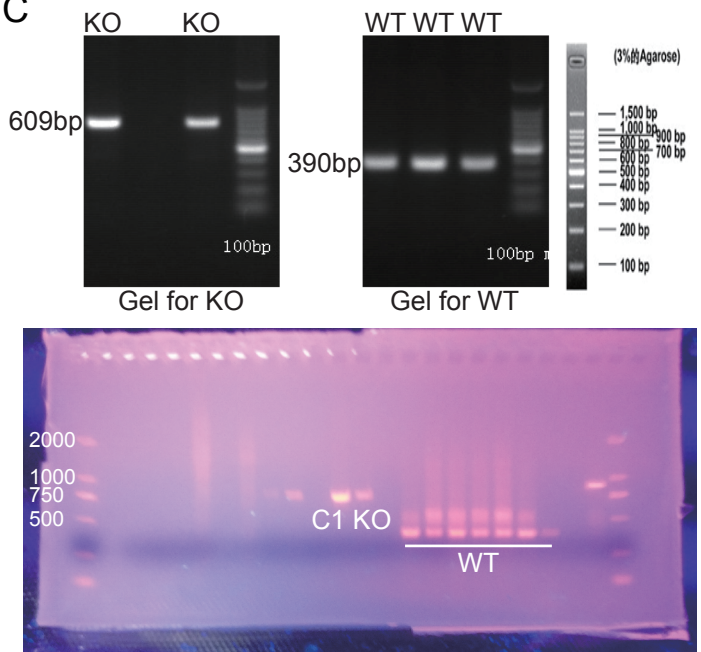

D

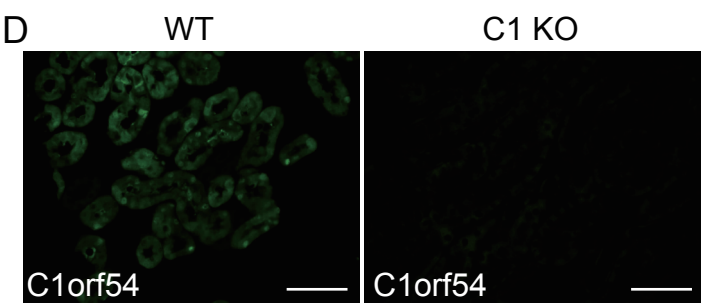

Supplement: Supplementary file 1 [file JCMM-22-4985-s001.pdf]
