# Supplementary material for: Tubular epithelial C1orf54 mediates protection and recovery from acute kidney injury
Source: J Cell Mol Med. 2018 Jul 12;22(10):4985–96. doi: 10.1111/jcmm.13765 (PMC6156286; doi:10.1111/jcmm.13765)
Supplement: Supplementary file 2 [file JCMM-22-4985-s002.docx]

**Supplemental Material**

**Supplemental figure legend**

**Supplemental Figure 1. C1orf54 expression in small intestine and generation of C1orf54-knockout mice**

(**A**) Immunohistochemical analysis of C1orf54 expression in small intestine. (**B**) Strategy for generating C1orf54-flox mice. (**C**) Mouse-tail DNA genotyping of wild-type (WT) and C1orf54-knockout mice. (**D**) Immunofluorescent staining of C1orf54 in kidney from wild-type (WT) and C1orf54-knockout mice. Scale bar, 100 μm.
